# Influence of EEG References on N170 Component in Human Facial Recognition

**DOI:** 10.3389/fnins.2019.00705

**Published:** 2019-07-11

**Authors:** Yi Wang, Hua Huang, Hao Yang, Jian Xu, Site Mo, Hongyu Lai, Ting Wu, Junpeng Zhang

**Affiliations:** ^1^Department of Medical Information Engineering, College of Electrical Engineering, Sichuan University, Chengdu, China; ^2^Department of Magnetoencephalography, Nanjing Brain Hospital Affiliated to Nanjing Medical University, Nanjing, China

**Keywords:** electroencephalograph reference, ERP, N170, facial recognition, statistical parametric scalp mapping, REST

## Abstract

The choice of the reference electrode scheme is an important step in event-related potential (ERP) analysis. In order to explore the optimal electroencephalogram reference electrode scheme for the ERP signal related to facial recognition, we investigated the influence of average reference (AR), mean mastoid reference (MM), and Reference Electrode Standardization Technique (REST) on the N170 component via statistical analysis, statistical parametric scalp mappings (SPSM) and source analysis. The statistical results showed that the choice of reference electrode scheme has little effect on N170 latency (*p* > 0.05), but has an significant impact on N170 amplitude (*p* < 0.05). ANOVA results show that, for the three references scheme, there was statistically significant difference between N170 latency and amplitude induced by the unfamiliar face and that induced by the scrambled face (*p* < 0.05). Specifically, the SPSM results show an anterior and a temporo-occipital distribution for AR and REST, whereas just anterior distribution shown for MM. However, the circumstantial evidence provided by source analysis is more consistent with SPSM of AR and REST, compared with that of MM. These results indicate that the experimental results under the AR and REST references are more objective and appropriate. Thus, it is more appropriate to use AR and REST reference scheme settings in future facial recognition experiments.

## Introduction

Electroencephalogram (EEG) with excellent temporal resolution is useful for determining the stages of vision-evoked information processing within the brain and plays an important role in illustrating the brains response to visual stimuli. Since the N170 component of the event-related potential (ERP) is very sensitive to configural information of the face, N170 has been regarded by researchers as a marker of face-sensitive processing (Bentin et al., 1996; Eimer, 2000). That is to say, N170 latency is shorter and amplitude is larger in response to faces than in response to non-face objects. For this reason, the N170 component has been used as the main research object in many facial recognition experiments (Ziongolumbic and Bentin, 2007; Németh et al., 2014; Cao, 2015).

For ERP studies, reference is an unavoidable problem. Since there is no zero or neutral point on the human body that can be used as a reference electrode, the EEG records the voltage between the two scalp electrodes (Yao, 2001; Nunez and Srinivasan, 2006), thereby causing EEG measurements to inevitably be affected by any choice of reference location. The statistical parametric scalp mapping (SPSM), which is the scalp distribution of the significant statistical difference between two conditions, varied depending on the adopted references (Tian and Yao, 2013). The previous study showed that the voltage waveforms, power spectra, EEG coherence, and SPSM may change for different references because of their dependence on the choice of reference (Qin et al., 2010). Even in the same experiment, different references will lead to different EEG distributions, which in turn will lead to different experimental conclusions (Chella et al., 2016). This means that the use of a suitable reference electrode, which is closest to zero potential, is critical for obtaining accurate EEG recordings.

In order to reduce the effect of reference selection and provide a relatively neutral reference, at least with respect to the signal of interest, researchers have proposed different reference schemes, such as mean mastoid reference (MM), average reference (AR), vertex reference (Cz), and left mastoid reference (LM) (Yao, 2001). However, as far as current reference standards are concerned, there is no universally accepted reference scheme, with different laboratories and research fields using different reference electrodes. In ERP research, the most popular reference electrode scheme is AR. This is because AR uses the average of all electrode channels as a reference and is unbiased to any one electrode position. Nevertheless, some studies (Yao, 2001; Yao et al., 2007; Kayser and Tenke, 2010; Qin et al., 2010) have confirmed that some unknown false fluctuations may inevitably be induced by current popular references, thereby altering the true EEG information. Using one channel of scalp recordings as a reference will introduce their own physiological dynamic interference signal into the EEG, which will affect the EEG signals from both time and space. The AR, Cz, and MM reference methods will lead to significant distortion in the scalp power distribution and scalp network structure (Yao et al., 2007; Nunez, 2010; Qin et al., 2010). Under the MM reference scheme, the scalp power distribution actually significantly shifts to frontal and superficial positions (Yao et al., 2005). A zero or neutral potential point is the ideal choice for solving the inherent problem of using body surface points as a reference (Nunez et al., 1997). Theoretically, a point far away from the source within the brain can serve as an ideal reference electrode point. Based on these considerations, Yao published a seminal paper for zero reference in 2001 (Yao, 2001) and established the unique method Reference Electrode Standardization Technique (REST), which approximately transforms EEG data recorded with a scalp point reference into recordings taking an infinity point as reference. The superiority of REST over the currently popular AR and MM were repeatedly confirmed in studies that followed. In this study, we wanted to figure out the optimal electroencephalogram reference electrode scheme for the ERP component related to facial recognition.

The goal of this study was to objectively compare ERP N170 components under three different references in an attempt to determine the most suitable reference electrode for facial recognition experiments. We addressed this by comparing the ERP components referenced by AR, MM, and REST. Specifically, the N170 latency and amplitude were compared and analyzed via ANOVA; then, the SPSM of N170 was calculated. Additionally, in order to confirm the topological results, we calculated the activity source location of the cerebral cortex under different stimulation conditions and obtained the difference between the two stimuli. Then, this difference was projected back onto the scalp to obtain the corresponding EEG topographic map, serving as the standard to compare the performance of different reference electrodes. Finally, we compared the cortical source imaging with the SPSM of each reference condition, hoping that additional evidence for differences may be shown by the imaging results.

## Materials and Methods

### Participants

Our study used EEG data made available publicly online as part of the A multi-subject, multi-modal human neuroimaging dataset on Open fMRI^1^ (Wakeman and Henson, 2015). Nineteen healthy participants with normal or corrected-to-normal vision were recruited from the MRC Cognition and Brain Sciences Unit participant panel. But only 16 participants (9 male, 7 female; age range of 23–31 years; and mean age of 26) with higher data quality were analyzed in the experiment. All participants were Caucasian except for one Asian participant who had spent many years in the United Kingdom. Written informed consent was obtained from each participant prior to and following each phase of the experiment. Participants also gave separate written consent for their anonymized data to be freely available online. All materials and procedures were approved by the Cambridge University Psychological Ethics Committee.

### Stimuli

**FIGURE 1:**
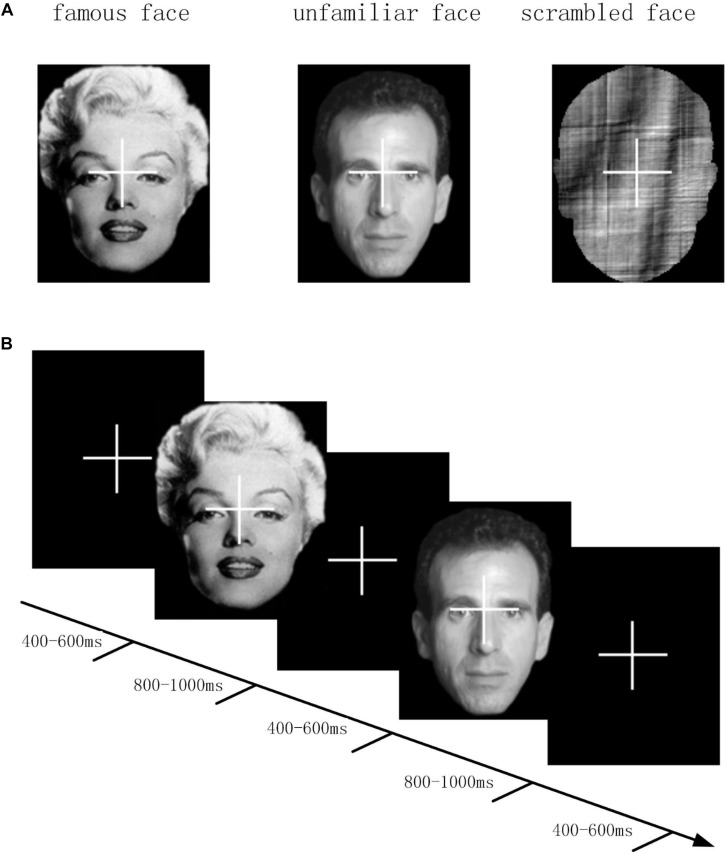
**(A)** Three different stimulus categories (famous face, unfamiliar face, and scrambled face) were gray scaled. **(B)** Timeline of one trial of EEG experiment. At the beginning of each trial, a fixed cross was presented for 400 to 600 ms. After the fixed cross, a stimulus image was presented for 800 to 1000 ms.

The experimental stimuli consisted of two categories: face image stimuli and non-face image stimuli (Figure 1A). The face image stimuli comprised of two sets of 300 gray-scale photographs for a total of 600 photographs. One set was of recognizable famous people and one set was of non-famous people who were unfamiliar to participants. Pictures of men and women were in equal proportion, each accounting for half. The famous faces were selected on the criteria that they were easily recognizable to the majority of British adults. Unfamiliar faces were approximately matched to famous faces in terms of age and sex so as to form two sets of 300 faces each. All images were cropped to show only the face. Additionally, the photos included a wide range of hairstyles, expressions, and orientations. The second stimulus category was non-face images, which consisted of scrambled face images that were generated from either the famous face images or unfamiliar face images. To be specific, they were scrambled by taking the 2D-Fourier transform of the face images, permuting the phase information, and then inverse-transforming them back into the image area. Lastly, in order to match the approximate shape and size of the original face images, the scrambled face images were cropped to a shape created by the combination of one famous face and one unfamiliar face.

### Procedure

During the experiment, participants were seated in a room that was well-lit, quiet, and magnetically shielded. Stimuli were projected onto a screen against a dark background with a visual angle of 3.66° × 5.38° and viewing distance of approximately 1.3 m. Participants were presented with sequences of experimental stimuli. To ensure attention to each stimulus, participants were asked to press one of two keys with either their left or right index finger as quickly as possible, without making any errors, when they saw the stimuli. They were instructed to press the left key if they thought the images were “more symmetric” than average and the left key if they thought the images were “less symmetric” than average. The reason for choosing this task was that it could be performed equally well on both face and non-face stimuli.

Each trial began with presenting a fixed cross for 400 to 600 ms, after which the experimental stimuli were superimposed against the fixed cross for a random duration between 800 and 1000 ms. Three stimulus conditions of famous face, unfamiliar face, and scrambled face were presented with equal probability in random order. Single experimental length was set to 7.5 min and each participant conducted six data acquisition experiments. Participants were required to fix their vision on the center of the image space throughout the experiment, as well as try not to blink during the cross-hair or stimulus. The timeline of an experimental trial is demonstrated in Figure 1B. Due to technical reasons, the incomplete data of three participants was removed.

### Data Recording and Preprocessing

EEG signals were recorded using a 70 channel Easycap EEG cap and stimulate (based on the EC80 system here^2^). The scalp electrode sites were Fpz, AFz, Fz, FCz, Cz, CPz, Pz, POz, Oz, Iz, FP1/2, AF3/4/7/8, FC1/2/3/4, FC, FT7/8/9/10, C1/2/3/4/5/6, T7/8, CP1/2/3/4/5/6, TP7/8/9/10, P1/2/3/4/5/6/7/8/9/10, PO3/4/7/8/9/10, and O1/2/9/10. A 3D digitizer (Fastrak Polhemus Inc., Colchester, VA, United States) was used to record the locations of the EEG electrodes, HPI coils, and approximately 50–100 “head points” around the scalp, relative to three anatomical fiducials (the nasion and left and right pre-auricular points). The EEG reference electrode was placed on the nose and the common ground electrode was placed at the left collar bone. Two sets of bipolar electrodes were used, with one set measuring the vertical and horizontal electro-oculograms (VEOG and HEOG) and the other set measuring the electro-cardiogram (ECG) of the left lower rib and right collarbone. Trials with blinks and eye movement were rejected on the basis of EOG. Data was acquired at an 1100 Hz sampling rate with a lowpass filter at 350 Hz and no high-pass filter.

The continuous data in each run was first epoched from −500 to +1,200 ms around the onset of each stimulus followed by manual detection of bad EEG channels. Then data was referenced to the AR based on the average off all electrodes, the MM, and the REST reference using the REST software. Both the AR and MM were conducted using Brainstorm in MATLAB, and the REST was conducted by the *rest_refer* function from www.neuro.uestc.edu.cn/rest (Dong et al., 2017). Then, all the AR, MM, and REST reference testing was conducted off-line to generate three long-term EEGs. These long-term EEGs of each electrode were firstly filtered through a band pass filter (0.5–32 Hz) and then segmented into epochs from −100 to 800 ms around the onset of each stimulus. Any epochs that contained more than ± 100 μV VEOG or HEOG potential were rejected as artifacts. EEG epochs were sorted according to stimulus conditions and were averaged for all subjects to compute the ERP. The baseline correction was conducted within the time window of −100 to 0 ms. In the present study, we chose three conditions (looking at famous face stimuli, looking at unfamiliar face stimuli, and looking at scrambled face stimuli) for which to compare the three reference schemes (AR, MM, and REST) with respect to temporal-spatial differences in the scalp voltages induced by different stimuli. The preprocessing of the EEG data was conducted by the brainstorm toolbox functions in the MATLAB environment.

### Data Analyses

Our research goals focused on exploring the impact of different reference electrode schemes on ERP data and determining the optimal electroencephalogram reference electrode scheme for ERP signals related to facial recognition. In present study, because the EEG data induced by famous faces and strange faces are very similar in latency and amplitude and the difference of N170 component between the unfamiliar faces and the scrambled faces seems larger, we chose to analyze the latter. The N170 is a negative component evoked in the occipital temporal lobe region between 150 and 210 ms after visual stimulation, with the amplitude reaching a maximum at about 170 ms after stimulation. Latency and amplitude of N170 at channel EEG065 (the label of EEG065 is PO10) close to the temporo-occipital was chosen as the main research objects. Previous studies (Maurer et al., 2002; Balas and Stevenson, 2014; Lueschow et al., 2015) have shown that there is a significant difference in amplitudes of N170 produced by different types of stimuli. A two-way repeated-measure was conducted to analyze the latency and amplitude of N170 induced by unfamiliar face and scrambled face stimuli under AR, MM, and REST references in order to investigate which reference scheme is most proper. Factors involved in the analyses were reference type (three levels: AR, MM, and REST) and stimulus type (two levels: unfamiliar face and scrambled face). The null-hypothesis rejection level was set at 0.05. Statistical analyses were performed with IBM SPSS Statistics 20 (SPSS Inc., Chicago, Illinois 60606).

However, only considering the statistically significant differences of N170 latency and amplitude was not enough. Besides this, we also made further topological analysis of scalp potentials. First, all the ERP epochs induced by two types of stimuli were averaged, respectively based on AR, MM, and REST reference to obtain the ERP of each reference. Then, N170 components for different reference conditions were extracted. Finally, the scalp N170 topographic map was obtained. Topographic maps can show cortical activity level in units of uV. In addition, we performed the paired *t*-test of N170 amplitude between unfamiliar face and scrambled face conditions for each electrode scheme. The parameter was set as *p* < 0.01 in order to have statistically significant difference for the correction method for the FDR. The above processes were performed for the data collected with each reference scheme. In addition, the correlation of SPSM results under three references was calculated. Such analysis sought to investigate whether the scalp distribution would be affected by reference scheme selection.

To further confirm the distribution of SPSM, minimum norm (MN) algorithm was used to illustrate the distribution of source activity for different stimuli. Firstly, the 3-shell sphere head model (conductivities of the Brain, Scalp and Skull is 0.33, 0.0042, 0.33, respectively) of each participant was calculated, with a total of 15,003 sources (voxels) distributed around the cerebral cortex in the head model. In the source calculation process, the parameter of Regularize noise covariance is set to 0.02, the parameter of Signal-to-noise ratio is set to 3.00. The source location result shows a threshold set to 35%. Secondly, we reconstructed the source activities of averaged ERP induced by unfamiliar face stimulus and that induced by scrambled face stimulus. Thirdly, a 20 ms time window around N170 peaks in source space for the unfamiliar face and scrambled face were intercepted and averaged, and we calculated the activity intensity of the bilateral occipital lobe region of the brain induced by unfamiliar faces and scrambled faces. Finally, the averaged source activity difference obtained from the last step were projected back into sensor space to visualize the topographic map, which reflects the differences in underlying source activity. All the source reconstruction of individual ERP was performed by Brainstorm in a MATLAB environment.

## Results

### ERP Measures

For the N170 amplitude, a repeated-measure ANOVA with two factors (Reference: AR, REST, and MM; Stimulus: Unfamiliar face and Scrambled face) was performed, significant main effects of reference (*F* = 6.66, *p* = 0.04,$\eta_{p}^{2}=0.31$) and stimulus (*F* = 44.52, *p* < 0.001,$\eta_{p}^{2}=0.74$) were separately observed. The interaction effect between reference and stimulus was non-significant (*F* = 3.23, *p* > 0.05,$\eta_{p}^{2}=0.17).$ For the N170 latency, non-significant main effects of reference (*F* = 2.582, *p* > 0.05,$\eta_{p}^{2}=0.14\;$) and significant effect of stimulus (*F* = 11.01, *p* = 0.046,$\eta_{p}^{2}=0.42$) were separately observed. The interaction effect between reference and stimulus was non-significant (*F* = 3.28, *p* > 0.05,$\eta_{p}^{2}=0.18$).

After that, we performed one-way ANOVA for N170 amplitude at electrode EEG065, calculated for the three references separately. The corresponding F- and *P*-values for different references described in Table 1.

**TABLE 1:** Comparison of N170 latency and amplitude among the AR, MM, and REST references.

		**AR**			**REST**			**MM**	
	
**Variables**	**Mean**	**Standard deviation**	**F/P**	**Mean**	**Standard deviation**	**F/P**	**Mean**	**Standard deviation**	**F/P**
*Latency*									
Unfamiliar	167.20	2.32	23.89/0.001	167.15	1.83	8.35/0.01	166.97	1.85	15/0.029
Scrambled	172.94	1.86		171.18	2.59		171.30	2.62	
*Amplitude*									
Unfamiliar	−11.14	1.11	48.04/0.001	−9.07	0.76	43.77/0.001	−9.98	1.15	33.57/0.001
Scrambled	−7.11	0.93		−5.78	0.89		−6.27	0.91	

### Voltage Topographies and SPSM

**FIGURE 2:**
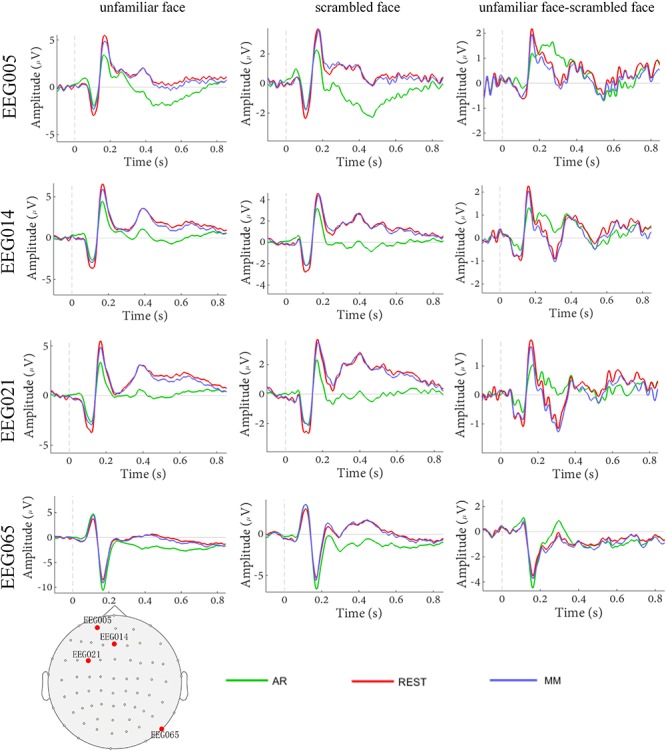
Comparison of face evoked ERP using three reference schemes (AR, MM, and REST). Waveforms of EEG005, EEG014, EEG021, and EEG065 ERP evoked using two stimulus conditions (unfamiliar face and scrambled face).

**FIGURE 3:**
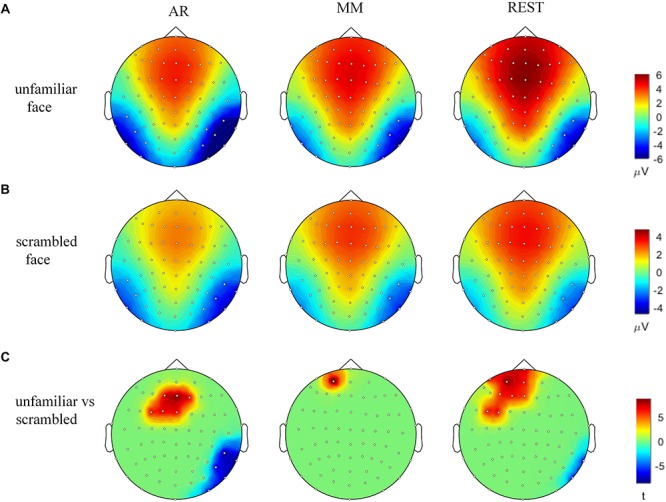
Voltage topographies and SPSM of N170 peaks. **(A)** Voltage topographies induced by unfamiliar face stimulus. **(B)** Voltage topographies induced by scrambled face stimulus. **(C)** SPSM (unfamiliar face vs. scrambled face), *p* < 0.01.

The waveforms of the ERP at channel EEG005, EEG014, EEG021 and EEG065 and the channel locations were illustrated in Figure 2. Figure 3 presents voltage topographies (unfamiliar face shown in Figure 3A, scrambled face shown in Figure 3B) and SPSM (shown in Figure 3C) of the N170 for each of the AR, MM, and REST reference schemes. As shown in Figure 3A, the distributions of the voltage topographies for unfamiliar face are similar among the three different references. Figure 3B illustrates that the voltage topographies for the scrambled face also show similar scalp distributions with the three different references. The only difference is a constant induced by the different references, which does not disturb the spatial distribution of the voltages (Pascual-Marqui and Lehamann, 1993; Geselowitz, 1998; Yao et al., 2007). This is similar to how fluctuating sea water around a mountain can change the mountain’s height above sea level but not change its shape. As can be seen in Figure 3C, however, the SPSM between unfamiliar face and scrambled face show significant differences in the spatial distributions for different references: AR indicates anterior and temporo-occipital distribution, MM indicates anterior distribution, and REST indicates anterior and temporo-occipital distribution. In other words, the experimental effect might show a frontal and a temporo-occipital distribution (Figure 3C, left; AR), a frontal distribution (Figure 3C, middle; MM), and a frontal and temporo-occipital distribution (Figure 3C, right; REST). We calculated the similarity of the three reference SPSM results in Figure 3C. The similarity between AR and MM is 38%, the similarity between MM and REST is 39.77%, and the similarity between AR and REST is 83%. The similarity between the sum of AR and MM and REST is 85%. These results reveal that, although the distribution of voltage topography is reference free, the amplitude difference induced by a reference can affect the experimental results, thereby possibly altering the final conclusion (Tian and Yao, 2013).

### Source Results

**FIGURE 4:**
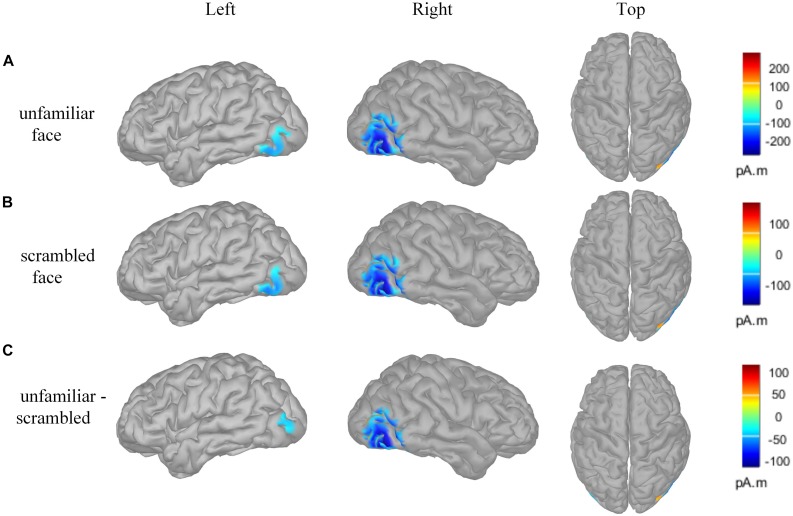
Brain activity source localization utilizing MN technology. **(A)** Unfamiliar face condition: bilateral posterior sources, and including bilateral temporo-occipital regions. **(B)** Scrambled face condition: bilateral posterior sources, and including temporo-occipital regions. **(C)** Source difference of unfamiliar face vs. scrambled face: bilateral posterior sources, and including temporo-occipital regions. In particular, the activation on the occipital cortex is stronger for the unfamiliar face stimulus than for the scrambled face stimulus, and the activation on the right occipital cortex is stronger than that on the left occipital cortex. The amplitude threshold of the above data was set to 35%.

**FIGURE 5:**
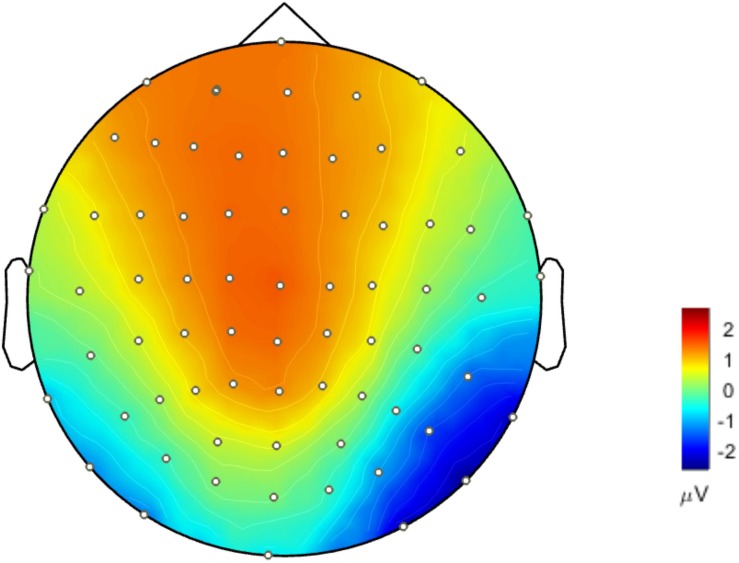
Topographical projection of the source activity difference of unfamiliar face vs. scrambled face (forward solution). Differences of N170 energy mainly concentrated around the occipital-temporal region and anterior lobe. The activity energy on the right occipital-temporal lobe was significantly stronger than that on the left temporal-occipital.

Figure 4 presents the cortical source distribution activated by unfamiliar face (Figure 4A) and scrambled face (Figure 4B), as well as the source difference between unfamiliar face and scrambled face (Figure 4C). These results indicate that the sources of activity are mainly located at the bilateral temporo-occipital cortex. As can be seen in Figure 4, regardless of whether subjects were looking at the unfamiliar face or scrambled face, the neural activities elicited by the visual stimuli were somewhat similar. However, a prominent source difference between unfamiliar face and scrambled face was shown over the right hemisphere of the brain. Figure 4C shows that a stronger activation appeared at the temporo-occipital cortex for unfamiliar face than for scrambled face. Under the stimulation of unfamiliar face, the average activity intensity of the left occipital lobe in the N170 band is 15.45 pA.m, the activated area of the brain area is 15.33 *cm*^2^, and the average activity intensity of the right occipital lobe is 37.87 pA.m, the average activated area is 42.54 *cm*^2^. Under the stimulation of scrambled face, the activity intensity of the left occipital lobe in the N170 band is 10.40 pA.m, the activated area of the brain area is 24.84 *cm*^2^, and the average activity intensity of the right occipital lobe is 18.73 pA.m. The activated area of the brain area is 42.51 *cm*^2^. Figure 5 illustrates voltage topographical projection of the source difference between unfamiliar face and scrambled face. The result shows that the differences between the two stimuli are mainly distributed in the right temporal-occipital area and anterior area, as is consistent with AR and REST-based SPSM of N170 (far-left and far-right subplot of Figure 3C).

## Discussion

In this study, three reference schemes (AR, MM, and REST) were comparatively investigated using statistics and topographical projections in order to find the most appropriate reference selection for facial recognition experiments. The results of the SPSM experiment (Figure 3C) for AR and REST references, in comparison to MM reference, are closer to the source forward results (Figure 5) and more consistent with the results of previous experiments (Blank et al., 2014; Jones and Zinsmeister, 2014).

Previous studies have shown that N170 is not affected by gender (Ito and Urland, 2005), spatial frequency information (Holmes et al., 2005). Therefore, this study only considers the effect of facial configural information on N170 latency and amplitude. The statistical results show that unfamiliar face with rich configural information attracts a person’s attention more than scrambled face without configural information. Furthermore, the statistical results also show that the N170 evoked by unfamiliar face is marked by shorter latency and larger amplitude than the N170 evoked by scrambled face. In general, significant difference is shown not only in N170 latency but also in N170 amplitude for AR, REST and MM. Additionally, we confirmed that peak amplitudes at N170 electrodes were more pronounced when ERPs were referred to AR than when MM or REST reference schemes were applied (Rellecke et al., 2013). Furthermore, the results are in accordance with previous studies where higher N170 amplitudes over the right hemisphere were observed in response to a human face stimulus than in response to a scrambled face (Sagiv and Bentin, 2001; Luo et al., 2013; Rodríguez et al., 2014). N170 latency and amplitude are two important indicators of ERP analysis, with the N170 component being used in many published studies to gain insights into the time and functional properties of different aspects of facial recognition processing in the human brain. However, different reference schemes have resulted in different experimental results and correspondingly different psychological explanations (Eimer, 2011; Rossion and Jacques, 2011). The statistical results show that N170 latency and amplitude are influenced by reference scheme selection. The difference in reference scheme has larger influence on N170 amplitude than it does on N170 latency. Nevertheless, it cannot be concluded which reference type is more proper solely on the basis of whether it shows a difference between stimulus conditions.

From the SPSM results, the distribution patterns of the scalp potential topography under the AR, MM and REST references are different. Under the AR reference, the brain regions with large differences in activity intensity are the frontal and occipital temporal regions. Under the MM reference, the brain area with a large difference in activity intensity is the frontal lobe. Under the REST reference, brain regions with large differences in activity intensity are located in the frontal and occipital temporal regions. This result is consistent with previous research results (Mnatsakanian and Tarkka, 2004; Pang, 2006). In general, the results of REST seem to be more comprehensive.

In order to further determine which reference is more proper, analysis source activity was conducted. For EEG, the scalp distribution voltage pattern is not affected by reference, and the EEG inverse problem is independent of scalp reference (Pascual-Marqui and Lehamann, 1993). That is to say, for a given cognitive problem, the source distribution within the brain is fixed and unchanged no matter which reference scheme is adopted. In Figure 3, it is seen that the source activity is distributed in the occipital lobe region on both sides of the brain, and no signs of source activity distribution are seen in the frontal lobe and parietal lobe. There are two possible reasons for this: On the one hand, the frontal lobe and parietal lobe are the area of high-level neural activity in which the brain processes logical reasoning, emotion control, sensory perceptions, etc., After receiving visual stimuli, each person responds differently to visual stimuli, and some people who produce negative EEG produce positive In the EEG, the positive and negative charges generated cancel each other out, so after the average, and the threshold is relatively high, the prefrontal area shows the absence of the illusion of EEG activity on the source. On the other hand, the method used to reconstruct the underlying source distribution is MN, which will gives blurred distributions whose maxima correspond to the real source locations (Wagner et al., 2004).

Thus, for the proposed study, if the scalp potential distribution (Figure 5) projected back from source distribution differences between unfamiliar face and scrambled face (Figure 4C) (it equals to solve the forward solution based on source distribution differences) is most similar with the SPSM under a given reference scheme, then that scheme should be most proper for facial recognition experiments. Such a strategy can provide us with a relatively objective comparison to infer which reference scheme is more proper. In this study, we used MN to obtain the cortical equivalent current sources of N170 evoked by unfamiliar face and scrambled face. The N170 sources are mainly located in the occipital temporal lobe regions on both sides of the brain (Figures 4A,B). Difference of cortical sources (Figure 4C) is projected back onto the scalp to obtain the corresponding EEG topographic map (Figure 5), which is able to visually reflect real differences in source activity within brain. As can be seen in the derived EEG map, there were large differences for the two stimuli in the anterior and the right temporo-occipital areas, as is more consistent with the AR and REST based SPSM results than it is with MM-based SPSM results.

According to classical literature concerning the topic (Itier et al., 2006; Luo et al., 2010), the brain region associated with N170 is mainly located in the temporo-occipital lobe. In this study, the electrode selected for analysis was also located in this area. The MM reference, if assumed to be a virtual electrode located somewhere between the two mastoids, should also be close to this area since MM is the mean of both mastoid electrodes. Just as stated in the literature (Junghöfer et al., 2006; Rellecke et al., 2013), the closer the sites of recording and reference are located to each other, the less pronounced are the effects on ERP. The experimental effect under MM reference is influenced by this relationship and may explain why, compared to that based on REST and AR, the MM-based effect is less pronounced. In summary, AR and REST-based results could faithfully reflect the activity source inside the brain. However, MM-based SPSMs are not consistent with the derived map. Therefore, the MM reference is not recommended in facial recognition experiments.

In this study, it was found that using AR and REST reference schemes showed the approximately true latency, amplitude, and SPSM mapping of N170. Compared to those referenced by MM, the ERP obtained with AR and REST references can achieve more objective analysis results, which may more accurately reflect the source activity within brain. Overall, the choice of reference is a very important and fundamental issue. This study illustrates that, at least for studies related to facial recognition, AR and REST references are clearly superior to MM references.

## Ethics Statement

Written informed consent was obtained from each participant prior to and following each phase of the experiment. Participants also gave separate written consent for their anonymized data to be freely available online. All materials and procedures were approved by the Cambridge University Psychological Ethics Committee.

## Author Contributions

JZ and YW conceived the idea and designed the research. HY and HH developed the models and methods. TW performed the numerical calculations. YW and JZ interpreted the results and wrote the manuscript. JX, SM, HL, TW, and HH revised the manuscript.

## Conflict of Interest Statement

The authors declare that the research was conducted in the absence of any commercial or financial relationships that could be construed as a potential conflict of interest.

## Footnotes

1 https://openfmri.org/dataset/ds000117/

2 https://www.easycap.de/wordpress/
